# Mr. W. D. Dray, on Dr. Huggan's Opinions

**Published:** 1800-04

**Authors:** W. D. Dray

**Affiliations:** Hythe, Kent


### *To the Editors of the Medical and Phyſical Journal.*

Gentlemen,

THE utility of a publication like your Journal muſt be to every one extremely obvious; but if any new mode of practice recommended, is introduced, which is not the reſult of ample, or, I may ſay, true experience, the moſt dangerous conſequences are likely to accrue; hence, how neceſſary it is for every medical man, before he publiſhes to the world any new remedy, to be particularly exact in his diagnoſis between the diſeaſe in which it is employed, and other diſorders; and alſo moſt accurate in his obſervations as to the real effects produced on the ſyſtem by ſuch a remedy.

Some time ago, I peruſed a letter in the 8th Number of your excellent Journal, on the effects of venæſection and opium, written by Dr. Huggan, of the Weſt Kent regiment of militia, wherein he endeavours to prove, that the former remedy may be ſafely, and more ſuccefsfully, ſuperſeded by the uſe of the latter. Now, as no anſwer to this letter has yet appeared, I think it my duty to make ſome obſervations on it, particularly as the welfare and even the very exiſtence of the ſick perſon, in many inſtances, depend on the judicious employment of one of theſe two means.

The author, after pointing out the many advantages of a regimental ſurgeon, begins, by aſſuring us, that *where his practice or opinions differ from others, they are the reſult of experience only, he having no theory to ſerve.* But in proceeding a little further, the gentleman is ſo truly unfortunate as to hold out a theory, which he appears to make the very baſis of all his practice; for after aſſerting that venæſection is unneceſſary in any diſeaſe whatever (which I hope to prove is a mere aſſertion) he begs the queſtion, by ſaying, "If we grant that every deviation from the healthy ſtate denotes debility, either general or partial, ſurely whatever has a tendency to debilitate further, it is reaſonable to ſuppoſe, ought to be carefully avoided." This is a propoſition that, with certain reſtrictions, muſt always hold good; but to admit of it ſo generally as the gentleman ſeems to demand, would be throwing aſide thoſe diſtinctions ſo eſſentially neceſſary in many diſeaſes. It may be ſaid, at the beginning beginning of fevers, there is debility; but may there not be fuch a re-action, in many cafes of fever, as to fet afide the idea of debility? or, may there not, in the firft ftage, be fuch an oppref-fion from the feverity of the attack, as to have little or no re-action, and the cafe be thus miftaken for real debility, when large bleedings, and other evacuations, would give freedom to the circulation, and prevent the fatal confequences that fo fre-quently enfue?

I am led to make thefe queries from my own obfervations and experience in pneumonic complaints, having been called upon to attend fome military men, where almoft every patient attacked with the fever died, becaufe it was fuppofed to be of the typhoid kind, from the great apparent debility at the com-mencement; till it was proved, by a very liberal ufe of the lan-cet, that there was a moft violent inflammatory diathefis, and a want of re-action. For it was a fact, that few or none died afterwards, under the timely employment of the antiphlogiftic plan.

I am not the only one who is of opinion that we have many difeafes as oppofite in their nature as poffible, which of courfe require a contrary kind of treatment; however, I fhall only now allude to pneumonia, or inflammation of the lungs and pleura, and the typhus mitior of Dr. Cullen, or what is moft generally known under the appellation of low nervous fever. I will venture to affirm, that every practitioner of difcernment, who has feen thefe two affections in their moft exquifite ftate, cannot but have noticed, that in the former there exifts an evident increafed action, or energy of the vafcular fyftem (except in thofe inftances of oppreffion above mentioned); in the latter, fymptoms of debility from the very commencement, and through the whole of the diforder; therefore, as this is fo clearly the cafe, the moft rational method that I can conceive of treating inflammatory complaints, is by employing, inftead of ftimulants, means which diminifh the increafed excitement, fuch as venæfection, &c. and a rigid adherence to the anti-phlogiftic plan; not by adding fuel to the fire, fo clearly exem-plified in the adminiftration of opiates; for even blifters cannot be applied with any degree of fafety in violent pneumonic com-plaints, unlefs preceded by the above means (a fact well known to every accurate obferver), and much lefs can fuch a powerful ftimulant as opium be given; for, if the inflammatory diathefis is not fufficiently fubdued, they never fail to increafe the dyfpnœa and other fymptoms; but when judicioufly adminiftered in the latter ftages, when the expectoration is thin and copious, and the cough urgent, the happieft effects may be expected from them. Yes, daily obfervation convinces us that this practice will will as certainly anſwer, as the proper uſe of ſtimulants in real aſthenic complaints, where phlebotomy would be as indubitably injurious, as opium preſcribed for thoſe of the ſthenic kind.

This I conceive to be the opinion of the moſt celebrated phyſicians of the paſt, as well as preſent ages, and which has of courſe been ſubſtantiated by ſuch an unlimited and true experience, as ought to make any practitioner cautious in his attempts to eſtabliſh, as facts, whatever tends to overthrow a doctrine ſo confirmed.

I will readily concur with the author, that *a prevalence of any particular mode of practice is not a convincing proof of its utility, or even ſafety*; but, ſurely, the gentleman will allow others to be as careful in their obſervations as himſelf: indeed, a man muſt be uncommonly weak to perſiſt in a routine, which proves more detrimental than ſalutary; but a practice ſo eſtabliſhed by daily occurring facts (as that for which I am now arguing) will, I believe, never be ſubverted by one ſo truly incongruous as that which the gentleman has advanced.

The author will no doubt appeal to the caſes which have been publiſhed in ſupport of his aſſertion. I totally agree with him, that caſes, with their ſymptoms juſtly and accurately detailed, with the experiments made on them, beſides being productive of the greateſt benefit to mankind in general, are ſufficient to warrant the uſe of a particular remedy; but a few caſes only, ought not to ſatisfy any practitioner. I muſt take the liberty to ſay, that there does not appear to me a ſingle caſe in point, among the author's ſelection, by which his aſſertion can be aſcertained; nor has he been very minute in his details of the few caſes he has publiſhed; circumſtances that muſt render them very unſatisfactory. It is not improbable that the loſs of blood, in ſome of the caſes of contuſion, might have prevented the coming on of bad ſymptoms; though, indeed, in moſt of them, the injury does not ſeem to have been ſo great as to make one apprehend danger from opium; alſo, in the pneumonic caſe, the ſymptoms appear to have been ſo mild that a recovery might have happened as ſoon (or ſooner) without the uſe of it; perhaps the effects of the firſt doſe were counteracted by the union of the calomel with it, which might have acted on the bowels, and a catharſis have been produced, or the affection might have been more ſpaſmodic than inflammatory: the others were evidently ſpaſmodic, and, as ſuch, opium was certainly requiſite, particularly when in combination with a cathartic.

We have only now to conſider the latter part of Dr. Huggan's letter, relating to the opinion of Dr. Trotter, concerning ague, where the author again aſſerts, *that he is confident, blood-letting will never be found neceſſary.* But I muſt beg leave to differ differ from him alſo in this inſtance, and coincide with Dr. F. that in ſome inſtances venæſection is indiſpenſable. I had a caſe in point, not long ſince: A ſoldier, who after having had ſeveral returns of an ague, was ſeized with a moſt violent dry cough, from imprudent expoſure to cold, and which, during every hot fit, was attended with pain and difficulty in reſpiration; he was almoſt inſtantly relieved by a large bleeding; and what is worthy of remark, the bark appeared greatly to aggravate the cough previous to his loſing blood; but after it, a ſpeedy recovery from the uſe of the bark was the conſequence.

I now flatter myſelf the unprejudiced reader will do me the juſtice to acknowledge, that ſufficient arguments have been adduced to controvert the opinion of Dr. Huggan; an opinion in its nature ſo calculated to miſlead young and inexperienced practitioners, as to be productive of the moſt fatal conſequences. If what he ſuppoſes be true, that *every derangement of the ſyſtem depends on debility*, the neceſſary quantity of blood taken away in ſome inſtances muſt induce irreparable miſchief; but, on the contrary, we find the recovery to happen frequently much ſooner than any body, a priori, would imagine; I ſhall therefore conclude, with briefly mentioning two caſes in point. The firſt was that of a man labouring under pneumonia in the Clinical Ward of the Royal Infirmary at Edinburgh, and under that judicious and admirable practitioner, the preſent Dr. Gregory. This patient had loſt about a pound of blood, before his admiſſion, and the ſymptoms being very ſevere, Dr. G. adviſed a large bleeding, which greatly relieved the pain and dyſpnœa; but theſe ſymptoms returning, he loſt eighteen ounces more, with ſome relief. On the third day, they recurred as violent as before, and thirty-two ounces were taken away at a ſingle bleeding; the next morning Dr. G. thinking a repetition neceſſary, twenty-two ounces more were taken from him; ſo that, firſt and laſt, this man loſt above an hundred ounces of blood, which, according to the calculation of phyſiologiſts, was full (or more than) a fourth part of his circulating maſs. And after this treatment, to my great ſurpriſe, his pulſe remained full and ſtrong, even to the time of his diſmiſſal.

The other was a ſimilar caſe, which very lately fell under my own obſervation; this man loſt near eighty ounces of blood, and to which I almoſt entirely attributed his recovery.

A maxim of Galen's now preſents itſelf to my mind, viz. *Reaſon as a practitioner, and practiſe with reaſon.*

By inſerting the above in your next Journal, you will greatly oblige,

Gentlemen,

Your humble ſervant,

*Hythe, Kent, March* 8, 1800. W. D. DRAY.

